# Insights From Single Cell RNA Sequencing Into the Immunology of Type 1 Diabetes- Cell Phenotypes and Antigen Specificity

**DOI:** 10.3389/fimmu.2021.751701

**Published:** 2021-10-01

**Authors:** Stephanie J. Hanna, Danijela Tatovic, Terri C. Thayer, Colin M. Dayan

**Affiliations:** ^1^ Diabetes Research Group, Division of Infection and Immunity, School of Medicine, Cardiff University, Cardiff, United Kingdom; ^2^ Department of Biological and Chemical Sciences, School of Natural and Social Sciences, Roberts Wesleyan College, Rochester, NY, United States; ^3^ Wellcome Centre for Human Genetics, University of Oxford, Oxford, United Kingdom

**Keywords:** type 1 diabetes, scRNAseq, immunology, lymphocytes, TCR - T cell receptor, BCR - B cell receptor

## Abstract

In the past few years, huge advances have been made in techniques to analyse cells at an individual level using RNA sequencing, and many of these have precipitated exciting discoveries in the immunology of type 1 diabetes (T1D). This review will cover the first papers to use scRNAseq to characterise human lymphocyte phenotypes in T1D in the peripheral blood, pancreatic lymph nodes and islets. These have revealed specific genes such as IL-32 that are differentially expressed in islet –specific T cells in T1D. scRNAseq has also revealed wider gene expression patterns that are involved in T1D and can predict its development even predating autoantibody production. Single cell sequencing of TCRs has revealed V genes and CDR3 motifs that are commonly used to target islet autoantigens, although truly public TCRs remain elusive. Little is known about BCR repertoires in T1D, but scRNAseq approaches have revealed that insulin binding BCRs commonly use specific J genes, share motifs between donors and frequently demonstrate poly-reactivity. This review will also summarise new developments in scRNAseq technology, the insights they have given into other diseases and how they could be leveraged to advance research in the type 1 diabetes field to identify novel biomarkers and targets for immunotherapy.

## Introduction

It is widely accepted that in T1D “there remains a paucity of robust and accepted biomarkers that can effectively inform on the activity of T cells during the natural history of the disease or in response to treatment” (1). Furthermore, the phenotype and roles of autoreactive B cells in T1D have received less attention than T cells (2–4). Whilst flow and mass cytometry approaches have enabled many insights into cell phenotypes and antigen specificity in type 1 diabetes [reviewed (5)], they allow detection of a relatively small number of markers, limiting the potential to discover truly novel biomarkers. In turn this limits the ability to monitor the natural history of diabetes development and patient responses to immunotherapy. In addition, although a number of immunomodulatory agents are in clinical trials for type 1 diabetes, these are generally non-specific in their actions (for example targeting CD3 or CD20) (6), and there remains a need to identify and target pathways that are perturbed specifically in islet-antigen specific lymphocytes.

Traditional RNA sequencing involves taking all cells of interest, and combining their RNA in a single sample before sequencing. In contrast, single cell RNA sequencing isolates individual cells, either through sorting into wells, or using droplet based technology (7). Transcripts from each cell are barcoded (a unique molecular identifier is also added to each transcript to circumvent any amplification bias), before being combined for sequencing. This allows quantification of the expression of every gene in every individual cell, so that cell phenotypes and heterogeneity can be fully elucidated. Of particular interest to immunologists are scRNAseq methods that allow sequencing across the V(D)J region of TCRs and BCRs. This allows capture of the paired TCRα and β chains (or paired heavy and light chains of BCRs) which is key to determining antigen specificity (8, 9) and being able to reconstitute the receptor in a cell line or to express it as a secreted antibody. A single cell sequencing approach also avoids much of the bias of bulk RNAseq of receptors (10).

There are a variety of methods used for scRNAseq [reviewed (7)] although the 10x Genomics platform has come to dominate the field, due to the relatively large number of cells that can be sampled and options of combining, for example, protein expression and V(D)J sequencing with standard gene expression (GEX) data (11, 12). In parallel, there has been an explosion in techniques to deal with the vast quantity of data generated, perform quality control and extract meaningful findings (13).

However, scRNAseq also comes with a number of caveats. Firstly it is technically challenging and poor sample preparation can lead to doublet formation in a similar manner to that seen in flow cytometry, but in addition scRNAseq samples are susceptible to contamination with RNA from dying cells and the downstream clustering algorithms can also produce seemingly novel cell populations which are in fact artefacts (14). Secondly, the high cost can make it somewhat inaccessible and limit sample numbers and sizes. Lastly, it requires stringent statistical analysis to avoid type 1 errors, preferably backed up by follow up experiments to verify findings (13).

Nevertheless, scRNAseq offers an exciting opportunity to identify novel biomarkers that could be indicative of diabetes progression in at risk individuals, and allow real-time monitoring of clinical trials through tracking expression of specific immuno-receptor sequences and cell phenotypes. Furthermore, it has the potential to discover novel targets for immunotherapy of type 1 diabetes, through the identification of genes that are differentially expressed in islet-antigen specific lymphocytes.

## Using scRNAseq to Identify Biomarkers for Progression to Type 1 Diabetes and Phenotypes in T1D

scRNAseq’s potential is demonstrated in a paper by Kallionpää et al. They revealed that high IL-32 expression in PBMCs was strongly associated with seroconversion and progression to T1D, contributed mainly by activated, highly differentiated, T cells and NK cells. Interestingly insulin (INS), glucagon (GCG), and REG1A were found to be upregulated in T1D and AAB+ individuals in the bulk RNAseq of PBMC but not in scRNAseq (15). These genes are normally associated with the pancreas, but expressed at the mRNA level in whole blood and lymph nodes at much lower levels (www.genecards.org). For insulin in particular this wider expression is thought to be involved in peripheral maintenance of tolerance (16).

We can also glean insight into the immunology of T1D from scRNAseq studies of the pancreas, as in T1D these will include infiltrating immune cells. For example, the Vahedi group performed scRNAseq of human pancreatic islet cells and found particular enrichment of antigen-presenting cells and macrophages in T1D (17). In a strong replication of Kallionpää et al.’s findings, an analysis of differentially expressed genes (DEG) in immune cells between healthy and type 1 diabetes pancreas samples identified REG1B, REG1A, INS and REG3A and IL-32 as highly differentially expressed (17). As with INS, GCG and REG1A, REG1B and REG3A are highly expressed in the pancreas but at lower levels in the blood and lymph nodes. Furthermore REG genes are reported to be upregulated in the pancreas not only in people that have T1D, but also those who are autoantibody positive (18). They are upregulated in inflammatory conditions and are thought to be important in the survival of beta cells in T1D (18). An alternative explanation for the association of these RNA transcripts with immune cells is that RNA transcripts from dying beta cells are contaminating other cell types during the scRNAseq process (19). A similar scRNAseq analysis of the NOD mouse pancreas has also been conducted (20) and scRNAseq has been used to characterise hESCs differentiating into beta cells (21). Studies using scRNAseq to investigate the human pancreas and T1D are summarised in **Table 1**.

**Table 1:** human scRNAseq gene expression studies relevant to type 1 diabetes.

Paper	Tissues	Antigen receptors	T1D status	Citation	Notes
Fasolino et al.	Pancreas	no	Healthy donors, AAB+, T1D	(17)	
Kallionpää et al.	PBMC	no	Healthy donors, AAB+	(15)	Children <3 2/4 AAB+ rapidly developed T1D
Xin et al.	Pancreas	no	Healthy donors, T1D	(22)	All islet cells sequenced, but analysis of beta cells only
Chiou et al.	PBMC pancreas	no	Healthy donors, T1D	(23)	scATACseq of PBMCs and pancreas of healthy donors. Reanalysis of healthy donor and T1D islet scRNAseq (22)
Cerosaletti et al.	PBMC	TCRs	Healthy donors, T1D	(24)	Islet-reactive T cells
Fuchs YF et al.	PBMC	TCRs	Healthy donors, T1D	(25)	Only one T1D sample
Culina S et al.	PBMC	TCRs	Healthy donors, T1D	(26)	Sorted ZnT8 186-194 MMr+CD8+ T cells.
Heninger et al.	PBMC	TCRs	Healthy donors, children who later progressed to T1D	(27)	GAD65- and proinsulin-responsive CD4+ T cells, limited genes sequenced in scRNAseq
Ahmed R, et al.	PBMC	TCRs, BCRs	T1D	(28)	Single donor
Hao Y et al.	Pancreas	no	Healthy children-older adults (29)	(12)	Combines multiple previous scRNAseq datasets to make a reference dataset and app, Azimuth
			Unspecified (30)		
			Unspecified (31)		
			Healthy controls and T2D (32)		
			Healthy controls and T2D (33)		
			Unspecified (34)		
			Healthy controls (35)		

Closely related to scRNAseq is scATACseq, whereby the DNA from individual cell nuclei is analysed to identify open or accessible chromatin regions and hence predict which genes are being expressed in each cell. Recently, Chiou et al. combined scATACseq with bulk ATACseq and scRNAseq approaches to link cis-regulatory elements (CREs e.g. gene promoters and enhancers) in peripheral blood cells and pancreatic cells with GWAS of diabetes risk (23). As would be expected this identified many CREs used in T cells and beta cells that had genetic variants associated with T1D susceptibility. For example CREs that controlled CTLA4 and CCR7 expression in T cells had variants associated with T1D. Importantly, this paper also identified CREs used in pancreatic cells that had polymorphisms associated with T1D risk, particularly those used in acinar and ductal cells. They were further able to map the T1D risk allele of rs7795896 to a CRE used in ductal cells. The risk variant was associated with decreased CFTR expression in ductal cells. Mutations in CFTR itself cause cystic fibrosis, frequently associated with pancreatic exocrine and endocrine abnormalities, but this is this first demonstration of a role for it, and may other genes expressed in the exocrine pancreas, in T1D pathogenesis. This paper also produced a reference map of single-cell chromatin accessibility from T1D-relevant cells from healthy donors (i.e. lymphoid, myeloid and pancreatic endocrine and non-endocrine cells). Interestingly scRNAseq of the human pancreas also identified multiple changes in gene expression in ductal cells in T1D (17). In particular expression of MHC Class II pathway and interferon alpha and beta pathway genes were increased. Other developments in the field of epigenetics of T1D and the interplay with environmental triggers [reviewed (36–38)] have also started to yield evidence of pathogenic roles for molecules such as BACH2, IL23A, IL6R and IL6ST in T cell function in T1D (39). It will be of great interest to see how our understanding of epigenetics in T1D develops at the single cell level.

## scRNAseq of TCRs

### Methods to Identify Antigen Specific T Cells

As discussed above, there are many advantages of single cell sequencing TCRs over bulk TCR repertoire sequencing. Before the advent of large scale scRNAseq, many people in the type 1 diabetes field appreciated the importance of sequencing immunoreceptors on a single cell basis and linking this to antigen specificity and affinity (40–42). As of 2017 there were 1655 clonotypes of known specificity for T1D autoantigens (41), a number which has increased substantially with the advent of higher throughput scRNAseq.

These have been identified through a numbers of methods. HLA class I or class II multimers may be used to select antigen specific T cells. This has the advantage of being able to select cells from the peripheral blood but is limited by HLA restriction and to known epitopes or mimotopes (43). In addition non-specific binding may yield false-positive TCRs. Alternatively peripheral blood T cells can be stimulated *in vitro* with islet peptide pools and selected on the basis of upregulation of activation markers, allowing wider specificities and HLA compatibilities, but with the risk of bystander activation again resulting in false negatives. A third approach is to sample T cells directly from the pLN or pancreas, where islet-specific T cells will be massively enriched. These cells can then either be stimulated *in vitro* with peptide pools the TCRs re-expressed *ex vivo* to determine specificity. Alternatively, TCR sequences can be compared to those in the literature known to be islet antigen specific.

### Diabetes Autoantigen- Specific Paired TCRs in the Peripheral Blood

Eugster and colleagues performed an heroic effort to sequence paired TCRs from 1650 T cells that either bound a GAD tetramer or responded to GAD *in vitro*, by sorting single cells from the peripheral blood and performing plate based scRNAseq (44). GAD specific TCRs were highly heterogenous both within and between donors, with no shared TCRs between donors, although individual TCRα or TCRβ chains were often shared. Moreover, there was limited overlap between the TCRs identified by tetramer binding and T cell activation methods, indicating that epitope recognition and MHC usage by GAD specific TCRs was likely to be broad (44).

Cerosaletti et al. performed scRNAseq of islet-reactive TCRs from the peripheral blood (identified by *ex vivo* response to stimulation with islet-peptide pools). They found that T cells from T1D had higher numbers of identical CDR3, which had arisen by clonal expansion (i.e. T cells with identical TCRα and TCRβ chains, that have arisen by division of a parent cell), rather than convergent recombination (24). By re-expressing the TCRs in cell lines it was found that many of these TCRs in people with T1D were IGRP specific (24). It was further shown that donors with T1D had large clonal expansions of IGRP-reactive T cells in the peripheral blood and frequently used a specific shared TCRα chain, which was paired with different beta chains in each donor (45). Preferential usage of TRAJ53 and TRAV29 and TCRα chains bearing the motif SGGSNYKLTF were identified in single cell TCR sequencing of people with T1D. When a bulk sequencing approach was taken, a particular TCRα chain bearing this motif was highly enriched in the memory CD8+ T cells of autoantibody positive people and those with T1D compared to controls. Clones bearing the motif were also shown to directly kill IGRP- peptide bearing cells (45). T cell clones bearing both IGRP (24, 45) and hybrid insulin peptide- responsive TCRs are persistent over time (46). However, others have examined TCR repertoires in children progressing to diabetes and shared TCRs were not seen either between children or within the same child over time, indicating high diversity in the peripheral blood at this age (27).

### Diabetes Autoantigen- Specific Paired TCRs in the Pancreas and Pancreatic Lymph Nodes

Early work examined T cells from the pancreatic lymph nodes (pLN) of people with T1D and found high clonal expansions (47). Additionally there were many T cells that shared a TCRβ but had divergent TCRα. Many clonally expanded CD4+ T cells recognised insulin A1-15 in the context of DR4 (47). Pathiraja et al. grew out CD4+T cells from the pancreatic islets of a donor with T1D using anti CD3 and cytokine stimulation. Over 25% of these clones had TCRs that responded to proinsulin peptides restricted by HLA-DQ8 or the HLA-DQ8 transdimer and 30% of clones used TRBV5–1*01 (48). Whilst it is difficult to make direct comparisons to frequencies of islet-reactive T cells in the peripheral blood (26), it is clear that in the peripheral blood frequencies are much lower [around 0.01-0.05% of T cells in people with T1D (3, 44, 49)]. Most T cells isolated from the pancreas had unique clonotypes, whilst the majority of *in vivo* clonally expanded T cells were specific for proinsulin (48). It has also been found that ZnT8- reactive T cells were present at similar frequencies in the blood of healthy controls and people with T1D, but were enriched in the pancreas of the latter (26). Single cell sequencing of TCRs found a public ZnT8 specific CDR3B in the peripheral blood, and enriched in the pancreas of people with T1D, although the full TCRβ had divergent sequences due to different gene usages. ZnT8 reactive T cells also showed a bias towards TRBV19 and TRAV12-2 usage (26).

Seay et al. also found sharing of CDR3s between donors in the pancreas, with a TCRβ with homology to a known GAD reactive TCR found in 7/18 T1D donors (50). Furthermore a shared CDR3β chain was found in all people with T1D in the conventional T cell compartment, whilst in healthy controls it was predominantly in the Treg compartment (50). Interestingly TCR sequencing of GAD-responsive CD4+IL-13+ T cells from patients who had received injected GAD Alum found that they often used a highly public TCRβ (TCRα sequencing was not available) (51).

Direct capture of pancreatic T cells by the Nakayama group enabled single cell TCR sequencing and confirmed infiltration of proinsulin specific cells into the pancreas in T1D. Of the hundreds of TCRs sequenced, most were present only on a single cell, indicating a diversity of response even years after diagnosis. Clonal expansions were more likely in CD8+ T cells and these clones were found in multiple islets from the same donor, indicating *in vivo* migration. Furthermore, across three donors it was noted that whilst there were no identical TCRs, there were identical TCRα sequences and TCR subunits (52). When the TCRs were re-expressed, the B9-23 reactive TCRs isolated from the pancreas induced much higher IL-2 secretion compared to control B9-23 TCRs isolated from peripheral blood (52) which may indicate the former have a higher affinity for B9-23. Moreover, only the pancreas-derived TCRs were capable of a response to whole proinsulin presented by APCs (52).

Recently the Nakayama group has reconstructed individual TCRs from the pancreas of people with T1D. TCRs were selected for re-expression on the basis of clonal expansion or V gene usage previously associated with proinsulin C19-A3 specificity, and were found to recognise epitopes across preproinsulin and presented by a variety of MHC class II (53). Many TCRs recognised peptides in the region of B9-23, but others, (many from clonally expanded cells) recognised peptide right across from the signal peptide to the A chain. Furthermore, these TCRs recognised peptides in the context of diverse MHCII, although a preference was shown for DQ (53). Even with these constraints of the selection criteria in this study, this single cell approach showed a diversity of peptide and MHCII specificity that would have been missed using tetramers.

### New Avenues for scRNAseq of TCRs

Taken together, the evidence suggests that T cells with TCRs with higher affinity for diabetes autoantigens are more likely to be found in the pancreas than in the peripheral circulation. This represents a major challenge in T1D research as in other autoimmune diseases it is relatively straightforward to obtain samples from the site of autoimmune attack (54). For example in psoriatic arthritis, extraction of viable T cells directly from the affected joints enabled sequencing of paired TCR receptors and scRNAseq profiling of cells phenotypes (55). Even in the pancreas, clonal expansion is modest and whilst CDR3 sequences specific for many diabetes autoantigens are shared between donors, there is not yet evidence of truly public TCRs with identical TCRα and β chains. However, more widespread use of the VDJdb repository (56), IEDB (57) and the JDRF/nPOD CloneSearch might allow enriched motifs to become apparent across different experiments, although this would still be limited by HLA restriction. To further complicate the picture, scRNAseq has demonstrated that islet antigen –reactive T cells (24) and HIP reactive T cells in particular (58) sometimes express two TCRα chains, which are known to contribute to autoimmunity (59, 60).

### Phenotypes of Antigen-Specific T Cells: Combining TCR Sequencing With Gene Expression

Combining TCR sequencing (or selection based on autoantigen reactivity), with scRNAseq has the potential to give further insights into T cell function. This has not always been straightforward to demonstrate, for example analysis of IGRP- specific T cells from the peripheral blood did not show a distinctive gene expression (GEX) pattern in response to stimulation (25). Similarly scRNAseq of ZnT8 reactive cells from the peripheral blood of people with T1D showed similar GEX profiles to healthy controls, indicating that these peripheral T cells may not be playing a driving role in T1D, although T1D patients had higher expression of aryl hydrocarbon receptor (AHR) and aurora kinase A (AURKA) and lower expression of RORA (26).

The approach was more successful for Heninger et al, who demonstrated that proinflammatory responses to diabetes autoantigens were dominant in children who progressed to autoantibody positivity, whilst regulatory T cell responses were seen in those who didn’t (27). An algorithm based on gene expression in response to autoantigens enabled identification of which children would later progress to autoantibody positivity. As this group developed autoantibodies the GEX profiles of their CD4+ T cells changed towards increased expression of Th1 genes (27). These findings suggest that biomarkers of T1D susceptibility may allow identification of at risk children prior to seroconversion (61).

In addition, Cerosaletti et al. used islet peptide pools to stimulate T cells from the peripheral blood *in vitro* and characterised those that activated by scRNAseq. They did not observe a significant level of differentially expressed genes between healthy controls to those from people with T1D. However, when they focussed on cells from people with T1D that were highly clonally expanded (termed T1D-E cells), they found that these cells did have a unique transcriptional profile compared to islet reactive T cells from healthy controls or those from people with T1D that were not clonally expanded. T1D-E cells preferentially expressed genes associated with T cell activation and leukocyte differentiation (24). These experiments demonstrate how focussing on antigen specificity can enhance findings from scRNAseq.

## scRNAseq of BCRs

### Early Work to Determine Antibody Sequences

Early interest in autoantibodies in T1D, before the advent of scRNAseq, focussed on isolating GAD-specific B cells from people with T1D (62) and sequencing the BCRs from clones, which provided evidence that GAD autoantibodies have frequently undergone somatic maturation and are therefore from antigen-experienced B cells (63, 64). Similarly IA-2 specific antibodies sequenced from B cells from people with T1D also show evidence of somatic mutation (65–67). Anti-insulin antibodies have been sequenced from people with T1D, but may have arisen in response to injected insulin rather than endogenous insulin (68, 69) [reviewed (70)]. BCR sequencing combined with phenotyping of B cells has given great insight into B cell response in other autoimmune diseases (71) and in response to vaccinations (72) and in B cell lymphoma (73). Yet without the equivalent of a tetramer approach to identifying autoreactive B cells, phenotyping and characterisation of the BCR has lagged behind T cell research in T1D.

### Identifying Islet-Reactive BCRs in the Periphery, Pancreatic Lymph Nodes and Pancreas

Smith et al. developed an approach to isolate insulin reactive B cells from the peripheral blood of people with T1D, by flow cytometric sorting B cells that bound insulin conjugated to fluorescent tags. The authors were then able to sequence BCRs from individual cells. They demonstrated that insulin binding BCRs preferentially used JH6 gene segments which have previously been associated with autoreactivity and were biased towards use of positively charged amino acids in the CDR3 region (74). When re-expressed as antibodies, BCRs from anergic naive IgD^+^, IgM^−^ B cells demonstrated binding at levels thought to induce anergic B cell responses, whilst those from naïve B cells bound weakly and would likely be ignorant of insulin under physiological conditions (74).

scRNAseq has been used to characterise a novel lymphocyte population that express both TCRs and BCRs (28). It is suggested that these “dual expressors” (DE) are increased in frequency in type 1 diabetes and that in people with type 1 diabetes there is a public BCR which can stimulate insulin-reactive CD4+ T cells. However, this work remains controversial as others have been unable to replicate the enrichment of DE in T1D nor the specific public BCR sequence (75). This highlights the importance of good quality control at every step of scRNAseq experiments.

Isolation of CD19+IgG+ B cells from pancreatic lymph nodes from autoantibody positive donors and single cell sequencing of their BCRs demonstrated that no clonally expanded B cells were identified in the pLN. Antibodies were reconstructed from BCR sequencing, although very few of these were found to be specific for IA2 (none were specific for GAD and insulin was not tested) (76). Seay et al. also sorted and single cell sequenced the BCRs from pancreatic LNs. They found an enrichment of insulin binding motifs in pLN from people with T1D compared to controls (50). They also observed sequence overlap with autoreactive BCRs cloned from precursor (early immature) B cells from healthy donors previously published by Wardemann et al. (77). Wardemann et al. observed that not only are many BCRs from healthy donor precursor B cells insulin reactive, they are often also polyreactive to other autoantigens for example dsDNA, ssDNA or nuclear proteins (77). This polyreactivity has also been noted for both IgM and IgG insulin antibodies (68). Similarly Smith et al. demonstrated that all of their high affinity insulin binding BCRs were also reactive to LPS and chromatin (74). Polyreactive antibodies have been postulated to play a key role in the healthy immune system but are also implicated in a variety of autoimmune diseases (78, 79). It therefore appears that autoreactive B cells in T1D may span a wide range of phenotypes and the antibodies produced may often be polyreactive, however the limited number of studies make it difficult to draw firm conclusions.

## Future Perspectives on scRNAseq in Type 1 Diabetes

### New Single Cell Methods and Analysis Tools

scRNAseq is beginning to give fascinating insights into type 1 diabetes and new approaches may yield further discoveries. The first of these is spatial transcriptomics (80). In this technique, indexed oligos capture RNA from either fresh-frozen or formalin-fixed, paraffin-embedded tissue sections. This allows determination of gene expression on a level that is fast approaching single cell resolution. It has already been used to give insights into cell interactions in other diseases such as rheumatoid arthritis, where infiltrating leukocytes interact with target cells (81, 82). Spatial transcriptomics therefore has great potential to unravel lymphocyte interactions with beta cells in the pancreas and to give insight into different patterns of immune cell infiltration (2). In both type 1 (23) and type 2 diabetes (83) scATACseq has recently been used to link GWAS to epigenetic regulation of gene expression. New methodologies enabling combination of ATACseq, and CITEseq with scRNAseq in the same experiment will also contribute to the field (84). New analysis tools such as CellPhoneDB give the ability to map interactions between subsets of cells, based on DEG in scRNAseq datasets, which would allow identification of novel interactions between immune cells and beta cells in the pancreas (85, 86). This may become increasingly important as we begin to understand the role of beta cell stress and signalling in type 1 diabetes (6) as well as the involvement of other pancreatic cells in diabetes development (23). CellPhoneDB has been used to identify crosstalk between T cells and epithelial cells in ulcerative colitis (87) whilst in rheumatoid arthritis scRNAseq has revealed interaction pathways between B cells, fibroblasts and monocytes (88). Additionally, recent work from the Satija lab has brought together previously published scRNAseq datasets of pancreatic cells, including immune cells from healthy pancreatic samples (12), which will facilitate this type of analysis. This would be further enhanced were there a unified repository for T1D scRNAseq datasets, similar to those for COVID-19 (89).

### Technological and Analytical Approaches to Enhance Immunoreceptor Sequencing

We have seen how combining GEX with V(D)J sequencing has increased insights into T1D. The recent development of DNA barcoded multimers will allow now the determination of T cell antigen specificity in scRNAseq experiments (90, 91), whilst conjugation of whole proteins or large folded protein fragments to DNA barcodes will facilitate identification of antigen specific B cells (92).

Computational approaches to determine the likely interaction of an immunoreceptor with target antigen also have the potential to revolutionise the search for antigen specific TCRs and BCRs. Approaches such as tcrdist (93), GLIPH (94) and immune receptor network generation for BCRs (95) enable BCR and TCR sequences to be mapped and visualised, and those that differ by only one or two amino acids are assumed to target the same antigens. NetTCR (96) and TCRex (97) use neural networks and machine learning algorithms to cluster TCRs predicited to bind the same epitope. Recent advances such as ICON and TCRAI leverage scRNAseq technology along with oligo labelled dextramers. They utilise the paired TCRα and TCRβ transcripts to build libraries of antigen specific receptors, with a neural network to predict antigen specificity of TCRs. However, many of these approaches have been validated using viral or tumour antigens with well-defined epitopes. As we have seen in the sections above, whilst there definitely are peptide sequences from diabetes autoantigens that are widely recognised, the immune response also targets diverse sequences in different individuals. Furthermore, auto-antigenic TCRs tend to bind pMHC with lower affinity than TCRs targeting pathogens (98, 99) as high affinity self-reactive TCRs are generally deleted in the thymus. It is not clear how this lower affinity and lack of public TCRs may impact upon the usefulness of computational approaches for T1D.

### Biomarkers in Clinical Trials

In 2019, it was demonstrated that teplizumab could delay progression to T1D in high risk individuals (100). Further work confirmed a correlation between fold change in C-peptide and change in frequency of CD8+KLRG1+TIGIT+ T cells (101). scRNAseq of T cells from the clinical trials of teplizumab and other immunotherapies in T1D could offer an amazing opportunity to identify all biomarkers predictive of successful treatment. For example, scRNAseq studies have shown a variety of phenotypic markers induced *in vitro* with anti-CD3 antibodies in human PBMC, including a variety of interleukin receptors and markers of regulation and exhaustion including FOXP3, CTLA4, TNFRSF18, LAG3 and PDCD1 (102). In contrast, anti-CD3/CD28 stimulation of PBMC analysed with scRNAseq and CITEseq, showed phenotypes strongly associated with activation (although memory subsets also upregulated senescence) (103).

In the future, a deeper understanding of TCRs and BCRs has the potential to better quantify the risk of progression in autoantibody positive people. Monitoring the abundance and phenotypes of lymphocytes bearing specific CDR3 sequences or using specific V genes may also prove useful in monitoring immunotherapies, particularly antigen specific immunotherapies, where phenotypic changes in whole lymphocyte populations may not be so obvious (1, 104–106). In addition, BCRs also have the potential to be used in CAR-Treg cell immunotherapy as has been demonstrated in the NOD mouse (107).

### A Computational Approach to Move Beyond scRNAseq

scRNAseq has demonstrated its great potential to identify novel biomarkers both in T1D and other autoimmune diseases. However, it is both technically challenging and expensive. Therefore it is crucial that researchers should be able to translate findings from scRNAseq into more accessible diagnostic and monitoring tests, for example using standardised flow cytometry or qPCR panels as is starting to happen in cancer research (108, 109). Similarly in IBD, a machine learning approach allowed identification of a CD8+ T cell signature that could predict prognosis. These biomarkers were then developed into a commercially available whole blood qPCR test to facilitate personalised therapy (110).

In T1D, recent advances in computational analysis are beginning to allow discrimination of changes in cell subsets from bulk RNAseq. Mehdi et al. identified a peripheral blood transcriptomic signature that predicted autoantibody development (111). Of the DEG identified, many were associated with the ubiquitin-proteasome pathway, DC and T cell function and were potentially targets of drugs approved for other conditions (111). Xhonneux et al. (112), demonstrated from transcriptomics of whole blood that they could undertake “digital cytometry”, by mapping groups of genes back to cell types. Children who developed autoantibodies against insulin first, had a signature of increased NK cells and CD4+ memory T cells. In contrast, those who first developed autoantibodies to GAD had a reduced percentage of CD4+ memory T cells and NK cells, but increased activated NK cells. Harmonizome (113) was used to identify a G protein–coupled receptor, GPR171, predicted to control the immune signature found in IAA+ children (112). Adding gene expression information to predictive models, increased their accuracy in predicting later T1D development in children under 18 months (112).

## Discussion

The first papers to analyse lymphocytes from type 1 diabetes using scRNAseq have provided fascinating insights into phenotypes involved in driving the disease and identified new potential targets for immunotherapy, such as IL-32 (15, 17). scRNAseq of TCRs involved in T1D has revealed that autoantigen specific TCRs have a wide range of targets and that whilst single chains or CDR3s are often shared between donors, it is rare to see TCRs with both chains identical in multiple donors; hence public TCRs remain elusive. In the peripheral blood, diabetes autoantigen reactive cells do not always have distinct phenotypes in healthy donors compared to those with T1D (25, 26), and enrichment of islet reactive cells is much more pronounced in the pancreas and pancreatic lymph nodes. Combining TCR sequencing with T cell phenotyping has led to a deeper understanding of islet antigen-specific cells in the peripheral blood (24, 27). A key challenge, for which scRNAseq is ideally suited, will be to develop methods to identify which T cells in the periphery are truly involved in beta cell destruction, and which are simply able to bind islet antigen multimers but are not capable of either trafficking to the islets or contributing to beta cell killing. Looking to the future, it is clear that combining antigen specificity with scRNA phenotyping and new computational approaches, such as those that can give insight into interactions between islet cells and infiltrating lymphocytes, have the potential to revolutionise the field.

Relatively few papers have tackled single cell sequencing (or indeed bulk sequencing) of BCRs repertoires in T1D, but those available suggest that these BCRs have unique properties and are often polyreactive (50, 74, 77). New approaches to identify islet-antigen specific B cells with scRNAseq (92) will therefore have much to contribute to our knowledge of how islet autoantibodies develop and are involved in disease progression.

scRNAseq is ideally suited to identifying subtle phenotypic differences between cohorts and has demonstrated promise in identifying differentially expressed genes in people that will later progress to autoantibody positivity and T1D (27). Developing this approach will be key to identifying at-risk individuals and matching them to a novel immunotherapy that is appropriate for their stage and phenotype of disease (100, 101, 114, 115). Furthermore, new analytical approaches will enable scRNAseq findings to be translated into new immunotherapies and biomarkers to monitor effectiveness of those already in clinical trials.

## Author Contributions

SH wrote the first draft of the manuscript. All authors contributed to the article and approved the submitted version.

## Funding

SH is funded by the Diabetes Research and Wellness Foundation Professor David Matthews Non-Clinical Research Fellowship 2020. DT, TT, and CD are funded by Diabetes UK, JDRF, Diabetes Research and Wellness Foundation, Wellcome Trust ISSF 204824/Z/16/Z IND-B-SS003 and the Association of Physicians of GB and Ireland. Open access publication fees were provided by the Wellcome Trust via an open access funding grant to Cardiff University.

## Conflict of Interest

The authors declare that the research was conducted in the absence of any commercial or financial relationships that could be construed as a potential conflict of interest.

## Publisher’s Note

All claims expressed in this article are solely those of the authors and do not necessarily represent those of their affiliated organizations, or those of the publisher, the editors and the reviewers. Any product that may be evaluated in this article, or claim that may be made by its manufacturer, is not guaranteed or endorsed by the publisher.

## Abbreviations

AAB+, Autoantibody positive; DE cell, dual expressor cell reported to express both a TCR and a BCR’; DEG, differentially expressed genes; GEX, gene expression; scRNAseq, single cell RNA sequencing.
